# Research Questions and Priorities for Pediatric Tuberculosis: A Survey of Published Systematic Reviews and Meta-Analyses

**DOI:** 10.1155/2022/1686047

**Published:** 2022-02-07

**Authors:** Thomas Achombwom Vukugah, Vera Nyibi Ntoh, Derick Akompab Akoku, Simo Leonie, Amed Jacob

**Affiliations:** ^1^ICAP at Columbia University, Maputo, Mozambique; ^2^Atlantic International University, Honolulu, HI, USA; ^3^Elizabeth Glaser Pediatric AIDS Foundation, Yaoundé, Cameroon; ^4^Health Alliance International, Abidjan, Côte d'Ivoire; ^5^Department of Global Health, University of Washington, Seattle, WA, USA

## Abstract

**Background:**

Advancing a research agenda designed to meet the specific needs of children is critical to ending pediatric TB epidemic. Systematic reviews are increasingly informing policies in pediatric tuberculosis (TB) care and control. However, there is a paucity of information on pediatric TB research priorities. *Methodology*. We searched MEDLINE, EMBASE, Web of Science, and the Cochrane Library for systematic reviews and meta-analyses on any aspect related to pediatric TB published between 2015 and 2021. We used the UK Health Research Classification System (HRCS) to help us classify the research questions and priorities. *Findings*. In total, 29 systematic reviews, with 84 research questions, were included in this review. The four most common research topics in the area of detection were 43.33% screening and diagnosis of TB, 23.33% evaluation of treatments and therapeutic interventions, 13.34% TB etiology and risk factors, and 13.34% prevention of disease and conditions and promotion of well-being. The research priorities focused mainly on evaluating TB diagnosis by improving yield through enhanced in specimen collection or preparation and evaluating of bacteriological TB diagnostic tests. Other topics of future research were developing a treatment for TB in children, assessing the use of IPT in reducing TB-associated morbidity, evaluating the prioritization of an IPT-friendly healthcare environment, and providing additional guidance for the use of isoniazid in the prevention of TB in HIV-infected children.

**Conclusion:**

There is a need for more systematic reviews on pediatric TB. The review identified several key priorities for future pediatric TB research mainly in the domain of (1) “Detection, screening and diagnosis,” “Development of Treatments and Therapeutic Interventions,” and “Prevention of Disease and Conditions, and Promotion of Well-Being.” These domains are very relevant in the research component of the roadmap towards ending TB in children. It also will serve as an additional action in the WHO End TB strategy.

## 1. Introduction

Tuberculosis (TB) is a communicable disease and is among the top 10 causes of morbidity and mortality worldwide. In 2019, an estimated 10.0 million (range, 8.9–11.0 million) people fell ill with TB around the globe. As a result, there were 1.2 million (range, 1.1–1.3 million) TB deaths among HIV-negative individuals and an additional 208 000 deaths (range, 177 000–242 000) among HIV-positive individuals [1].

Traditionally, pediatric tuberculosis has been relatively neglected; although in recent years, there has been interest among the global health community to reduce childhood tuberculosis deaths [2]. In 2017, 55% of estimated children with TB (0–14 years) were not reported to the national TB programs [3]. Despite this enormous toll on health, the response to pediatric TB has been slow and underfunded, particularly in the area of research [4] which is critical to the development of new tools and approaches for elimination of pediatric TB by 2050 [5]. The need for advancing a research agenda designed to meet the specific needs of children is critical to ending the pediatric TB epidemic.

Research efforts regarding TB in children have focused mostly on applying existing tools to diagnose, treat, and prevent pediatric TB. Despite these aids, however, children have different needs than adults. For example, bacteriologic confirmation of Mycobacterium tuberculosis (Mtb) in children is often difficult to achieve because they are frequently unable to expectorate sputum for bacteriologic testing and often have paucibacillary disease that cannot be detected using sputum smear microscopy, culture, and/or molecular testing (e.g., Xpert (Cepheid, Sunnyvale CA, USA)) [6–8]. Further research is needed to better understand some of the basic characteristics of tuberculosis in infants, children, and adolescents, such as the immune response to infection and its associated biomarkers (regular changes in the body that can be reliably measured and indicate TB infection and disease), to help in the development of new tools [8]. Even though the Stop TB Partnership Child and Adolescent TB Working Group and Treatment Action Group have laid out a detailed list of research priorities for pediatric TB [9], there is still the need for a more focused research approach to adequately address the research concerns on pediatric TB. In addition, there is no systematic review on pediatric TB research priorities to guide future research in this field. We reviewed all the published systematic reviews and meta-analyses on pediatric TB (in all areas, including drugs, vaccines, and diagnostics), with the objective to (1) identify all systematic reviews and meta-analyses about any aspect of pediatric TB from 2015 to 2021 and (2) assess, compile, and rank the research priorities that were identified to help address gabs in subsequent pediatric TB research.

## 2. Methods

### 2.1. Searching

The databases MEDLINE, EMBASE, Web of Science, and the Cochrane Library were searched for reviews and meta-analyses on pediatric TB. The search strategy was developed in consultation with a medical librarian. The search focused on contemporary TB literature published between January 1, 2015, and February 28, 2021. The search strategy included the following keywords and MeSH terms: [‘Pediatric tuberculosis' (explode) OR ‘tuberculose pediatrique' (explode) OR ‘Mycobacterium tuberculosis'(explode) OR ‘childhood pediatric tuberculosis'(explode) OR ‘tuberculose chez l'enfant'(explode)] AND [‘meta-analysis'(explode) OR ‘meta-analyses'(explode) OR ‘review systematque'(explode) OR ‘systematic review'(explode)]. The search was limited to English and French.

### 2.2. Study Selection

Studies with focus on any aspect of pediatric TB were included. This involved systematic reviews and meta-analyses published in both English and French due to skill set of our research team. All reference lists were also reviewed to identify pertinent publications. We also consulted the websites of relevant government organizations and professional societies (e.g., WHO, Center for Disease Control and Prevention, European Center for Disease Control and Prevention, American Academy of Pediatrics, Health Protection Agency, UK Health Department, and the Union for relevant documents related to pediatric TB).

The first screening of the titles and abstracts was carried out by one reviewer (VAT). The same reviewer (VAT) then screened the full-text articles and decided on the final inclusion of the studies in the systematic review. In addition, a second reviewer (NV) independently searched, reviewed, and identified studies to be included in the review. Differences between the two reviewers were evened out after discussion.

### 2.3. Data Abstraction

EndNote X7 (Thomson Reuters Scientific Inc., Carlsbad, CA, USA) was used to enter all systematic review and meta-analysis results on pediatric tuberculosis, and duplicates deleted. Two authors (VAT and NV) reviewed titles and abstracts with the goal of removing publications that did not fit the criteria. The remaining papers were appraised independently after both authors reviewed 20% of the articles and demonstrated higher than 95% interrater reliability. In addition, on the study characteristics part of the data extraction form, a third reviewer (DA) independently extracted data for all included studies, and disagreements settled by a consensus.

### 2.4. Study Characteristics

Garrard's matrix strategy for abstracting was used to build a Microsoft Excel spreadsheet and abstract full texts of the remaining articles [10]. We extracted data from the text or online supplement of each included systematic review or meta-analysis. Information was collected on two main points: (1) the main focus of the systematic review and (2) questions and priorities identified for future research. The UK Health Research Classification System (HRCS) [11] developed by the UK Clinical Research Collaboration for the classification and analysis of all types of health research was used to determine the focus of the included studies as well as the focus of the research questions/priorities. In particular, the HRCS Research Activity Codes [11] were used to assign a category for the main focus of the studies and the research questions/priorities.

The main focus of each included systematic review was determined by extracting keywords from the title and abstract and matching them with the criteria developed by the HRCS. The codes were divided into eight major categories: (1) underpinning research; (2) aetiology; (3) prevention of disease and conditions and promotion of well-being; (4) detection, screening, and diagnosis; (5) development of treatments and therapeutic interventions; (6) evaluation of treatments and therapeutic interventions; (7) management of diseases and conditions; and (8) health and social care service research (see Table 1 for full description). These research categories were used in Tables 2 and 3, to provide an overarching framework for grouping pediatric TB research.

Each of the overarching eight HRCS code groups was further subdivided into five to nine subcategories with definitions for the type of research to be considered for that subcategory. For instance, the 5th code category known as “Development of Treatments and Therapeutic Interventions” includes nine subcategories: (6.1) pharmaceutical studies on clinical application and evaluation of pharmaceutical small molecules, therapeutic vaccines, antibodies and hormones in humans including phase I, II, II, and IV trials, monitoring response, outcome, drug resistance, and side effects; (6.2) studies on cellular and gene therapies evaluating cellular, tissue, and gene therapies in humans including small scale and pilot studies, phase I, II, III, and IV trials and applied delivery systems; (6.3) studies on medical devices including implantable devices, mobility aids, dressings, medical equipment and prostheses, validation of design, and postmarket surveillance; (6.4) studies on surgery evaluating surgical, obstetric, and dental intervention in humans including small scale and pilot studies, phase I, II, III, and IV trials and monitoring outcomes, side effects, and rejection; (6.5) studies on radiotherapy and other noninvasive therapies, evaluating interventions in human including scale and pilot studies, phase I, II, III, and IV trials and monitoring outcomes, and side effects; (6.6) psychological and behavioral study psychological and behavioral evaluating interventions in humans in clinical, community, and applied settings; (6.7) studies on physical testing and evaluation of physical interventions in humans in a clinical, community, or applied setting including physical therapies, physiotherapy, occupational therapy, speech therapy, dietetics, osteopathy, and exercise; (6.8) complementary studies which include all aspects of testing, evaluation, and provision of complementary approaches to conventional medicine in humans in a clinical, community, or applied setting including hypnotherapy, massage, acupuncture and homeopathy, issues relating to health and social services and healthcare delivery, attitudes and beliefs of patients, and healthcare professionals; and (6.9) studies on resources and infrastructure (evaluation of treatments) including the provision and distribution of resources related to clinical and applied therapeutic interventions and infrastructure support for clinical and applied research networks and trials, consortia, and centers. Using the main categories and the subcategories, we mapped the corresponding pediatric TB research areas found in the literature search (see Tables 1 and 2).

### 2.5. Quantitative Data Synthesis

Study characteristics were summarized using descriptive statistics. Measures such as total count, frequency, and proportion were used to summarize data. Data analyses were performed using Microsoft Excel 2010.

## 3. Results

**Figure 1:**
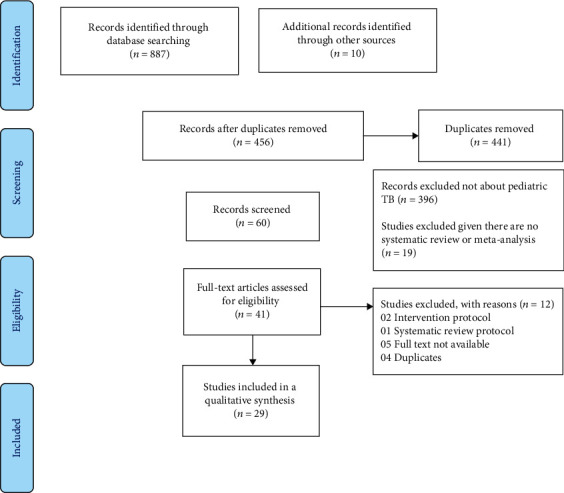
Flow chart of the study selection process.

A total of 897 records were identified through the electronic database search (Figure 1). An initial of 456 records was subjected to a preliminary screening of titles and abstracts with 415 excluded at the end of the process. The reasons for exclusion included are as follows: records not about pediatric TB (396) and studies were not systematic review or meta-analysis (19).

The full-text screening of published articles was performed on 41 records after which 12 records were excluded because they were either intervention protocol, systematic review protocol, duplicate, or full text not available. Overall, 29 systematic reviews were included in our analysis comprising 24 systematic reviews and meta-analyses exclusively on pediatric TB [12–35] and 5 systematic reviews and meta-analyses on adults and children [17, 36–39].

### 3.1. Characteristics of Included TB Systematic Reviews and Meta-analysis

The 29 reviews were published in 26 different journals. The majority of reviews (8, 28%) were published in journals with impact factors of five or less, and only three (10.3%) reviews were published in journals with a high impact factor (≥20). However, a large proportion of the reviews (18, 62%) were published in journals that did not have an impact factor. In addition, a majority (30%) of the main authors were from the United States. The remaining 70% of authors were from 12 different countries (Australia, Canada, China, England, Ethiopia, France, India, Iran, Italy, Korea, South Africa, and Switzerland).

Out of the 29 reviews, 28 (96.6%) self-identified as a systematic review or meta-analysis, which means that they used the term “systematic review” or “meta-analysis” in the title or abstract.

18 (62%) of the 29 reviews reported having a funding source, whereas only 3 reviews (10.0%) reported not being funded with 8 reviews (28%) reporting no funding status. Most of the reviews (27, 93%) included less than 50 studies in their review, and within those reviews, the majority had between 6 and 6,000 participants (23/29 (79%)).

## 4. Focus of TB Systematic Reviews

The main focus of each review was determined using the HRCS as previously described. The classification categories were subdivided into major tuberculosis research areas as described in Table 2. The four most common review categories, in decreasing order, were “Detection, Screening and Diagnosis” with 13/30 (43.33%) systematic reviews, “Evaluation of Treatments and Therapeutic Interventions” with 7/30 (23.33%) systematic reviews, “Aetiology” with 4/30 (13.34%) systematic reviews, and “Prevention of Disease and Conditions, and Promotion of Well-Being” with 4 of 4/30 (13.34%) systematic reviews.

In the “Detection, Screening and Diagnosis” main category, 8/13 (61.5%) of the reviews focused on the bacteriological diagnosis of TB in children, specifically the use of Xpert for TB diagnosis. The other two most common research areas were on the type of sample for TB diagnosis specifically the use of stool for diagnosing TB in children 2/13 (15.4%) and also TB screening in children (2/13 (15.4%)).

In the “Evaluation of Treatments and Therapeutic Interventions” main category, 3/7 (43%) of the studies focused on TB/HIV integration specifically on evaluating childhood TB treatment outcome and its association with HIV. The other two common research areas were on improving adherence to treatment for pediatric TB (2/7 (28.5%)) and Evaluation of Treatment outcome of TB in children (2/7 (28.5%)).

Among the “Prevention of Disease and Conditions, and Promotion of Well-Being” category, 3/4 (75%) of studies focused on chemoprophylaxis of TB in children, and one of the studies focused on barriers to the implementation of isoniazid preventive therapy (IPT) for tuberculosis in children.

Lastly, in the “Aetiology” category, 3/4 (75%) research studies focused on environmental or external factors associated with the cause, risk, or development of TB disease in children, and one study focused on mortality in children diagnosed with tuberculosis.

## 5. Research Priorities

Of the 29 reviews, 21 (72%) identified at least one research question or a research priority. Of these, 07 (33.3%) identified only one research priority, 05 (23.8%) two research priorities, 6 (28.5%) three, 13 (61.9%) four, and only one (4.6%) review identified more than five research priorities.

Table 3 shows the summary of research priorities by category, subdivision, and TB-specific research priority. The three major categories of research priorities/questions were “Detection, screening and diagnosis” responsible for 38/84 (45.2%) of all the identified research priorities, “Development of Treatments and Therapeutic Interventions” with 14/84 (15.5%), and “Prevention of Disease and Conditions, and Promotion of Well-Being” with 10/84 (11.9%).

In the most common category, “Detection, screening, and diagnosis,” the top research priorities were “Evaluating TB diagnosis by improving yield through improvements in specimen collection or preparation,” assessing the impact of GeneXpert on patient outcome (e.g., time to diagnosis, time to treatment, disease outcomes, health-system cost, and cost for families), assessing the rollout of GeneXpert and its implication on empirical TB treatment initiation, and applying transparent definitions for the certainty of diagnosis (e.g., confirmed tuberculosis and clinical tuberculosis in 15/38 (39.5) reviews). Other frequently cited pediatric TB research priorities were evaluating bacteriological TB diagnostic tests, 8/38 (21); assessing active case-finding for early diagnose of TB in children; assessing the development of screening algorithms and effective implementation of novel diagnostic tools; determining the incidence and prevalence of TB in children, 04/38 (10.5); assessing the role of Xpert in nontraditional tuberculosis settings (e.g., HIV clinics and malnutrition units); and evaluating the challenges of integrating Xpert into the health system, 3/8 (7.8%).

Within the category of “Development of Treatments and Therapeutic Interventions,” the main pediatric TB research priorities were developing a treatment for active and latent TB in children, 10/13 (76.9); monitoring of electrolytes (potassium and magnesium) and albumin in the management of TB in children, 2/13 (15.4); and standardized language to describe barriers to TB treatment initiation, within the TB research and advocacy community, 1/13 (7.7).

In the “Prevention of Disease and Conditions, and Promotion of Well-Being” main category, the major pediatric TB research priorities were assessing the use of IPT in reducing TB-associated morbidity; assessing the provision of preventive therapy to young children exposed to or infected with tuberculosis, 5/10 (50%); evaluating the prioritization of an IPT-friendly healthcare environment; providing additional guidance for the use of isoniazid in the prevention of TB in HIV-infected children, 4/10 (5/10); and evaluating BCG vaccine and HVI status for preventing TB in children, 1/10 (10%).

## 6. Discussion

To the best of our knowledge, this is the first systematic literature review on identifying research questions and priorities on pediatric TB worldwide. Systematic reviews and meta-analyses are widely acknowledged as a key component of the policy and guideline development process [40]. Systematic reviews often conclude by making suggestions for the direction of future research and thus could be a good source for identifying the most important questions for pediatric TB research. Our report collected descriptive information from all eligible systematic reviews and meta-analyses that were used to generate a list of research priorities in pediatric TB within the framework of the International Roadmap for Tuberculosis Research [3, 9].

Our systematic search showed that a limited number of systematic reviews were published on pediatric TB from 2015 to 2021. The findings of our review need to be interpreted within the framework of the Child and Adolescent TB working group which highlights priorities for future research initiatives in epidemiology, basic science, prevention, diagnosis, treatment, and operational research [9]. Our results show that the top three HRCS categories for a subsequent research priorities were “Detection, screening and diagnosis,” “Development of Treatments and Therapeutic Interventions,” and “Prevention of Disease and Conditions and Promotion of Well-Being.” TB prevention, diagnosis, and treatment were among the most important research priorities in both reviews. One possible reason why TB diagnosis research ranked high on our list could be that our review examined studies published between 2015 and 2021, a period when major advances were made in pediatric TB diagnostics, especially with the use of GeneXpert for TB diagnosis becoming a very popular subject of research [24, 41, 42], even though diagnosis of TB in children is still a challenge in pediatric TB. Additionally, there was an introduction of several WHO policies on pediatric TB diagnostics during the period [8, 43]. In addition, a similar study on adult TB suggested that “Detection, screening and diagnosis,” “Aetiology,” and “Evaluation of treatments and therapeutic interventions” are the main domain for research priorities [5, 44].

The research priorities determined mainly focused on evaluating TB diagnosis by improving yield through advancements in specimen collection or preparation and evaluation of bacteriological TB diagnostic tests. Many articles were cited on the need to assess the rollout of GeneXpert and its implication on empirical TB treatment initiation. In the subcategory of population screening, there were questions on assessing active case-finding for early diagnosis of TB in children and also determining the incidence and prevalence of TB in children. Other important topics were developing a treatment for active and latent TB among children, evaluating the use of IPT in reducing TB-associated morbidity, evaluating the provision of preventive therapy to young children exposed to or infected with TB, evaluating the prioritization of an IPT-friendly healthcare environment, and providing guidance for the use of IPT for the prevention of TB in HIV-infected children.

Although several systematic reviews identified areas for further research, the questions were often framed in a generic way, rather than in a highly focused manner with specific recommendations for action. Future TB systematic reviews will need to be more focused and propose very specific, answerable questions that are amenable to well-designed research studies.

A limitation of this study is the fact that only articles in English and French were screened limiting the number of research that were suitable for the inclusion in this analysis. We were also unable to contact authors or hand search journals as well as not including any unpublished literature. Due to its overarching and generic nature, the HRCS categories were at times nonspecific and difficult to match with specific areas of pediatric TB research. Furthermore, it was difficult to classify research priorities into narrow subdivisions since some research priorities could qualify for more than one subdivision. By categorizing research priorities into larger, predefined categories, we lost detailed information on individual research priorities. To remedy this, we condensed each priority and extracted the topic words from it. The topic words were then grouped to form the summary of repeated priorities/questions and the frequency calculated. Due to the poor overall quality of reporting of the systematic reviews, the findings may not be representative of the general output from the pediatric TB research community [45] given the likelihood of missing studies due to language barriers. Despite its limitations, the study certainly adds to our understanding of the need for a more systematic review on pediatric TB and also improving pediatric TB research agenda especially in the area of prevention and diagnosis.

## 7. Conclusion

In summary, our systematic review of published systematic reviews and meta-analysis on pediatric TB helped identify several key priorities for future pediatric TB research mainly in the domain of (1) “Detection, screening and diagnosis,” “Development of Treatments and Therapeutic Interventions,” and “Prevention of Disease and Conditions, and Promotion of Well-Being.” These domains are very relevant in the research component of the roadmap towards ending TB in children and adolescents. It also will serve as an additional action in the WHO End TB strategy. Our work was useful to describe the landscape of pediatric TB research and the overarching pediatric TB research themes arising from systematic reviews and meta-analyses conducted over the last 5 years. They are useful in indicating research priorities on areas that receive high attention, either due to recent scientific developments or increasing questions surrounding the advancement of knowledge in these very areas. They bring useful information to the broader, deeper, and more rigorously conducted process of international research agenda development. The study could be repeated including languages (more than two) as this could improve the study findings.

## Figures and Tables

**Table 1:** Description of the Health Research Classification System (HRCS).

Research activity code (description) and subcategory
(1) Underpinning research (research that underpins investigations into the cause, development, detection, treatment, and management of diseases, conditions, and ill health)
(1.1) Normal biological development and functioning
(1.2) Psychological and socio-economic processes
(1.3) Chemical and physical sciences
(1.4) Methodologies and measurements
(1.5) Resources and infrastructure (underpinning)
(2) Aetiology (identification of determinants that are involved in the cause, risk, or development of disease, conditions, and ill health)
(2.1) Biological and endogenous factors
(2.2) Factors relating to the physical environment
(2.3) Psychological, social, and economic factors
(2.4) Surveillance and distribution
(2.5) Research design and methodologies (etiology)
(2.6) Resources and infrastructure (etiology)
(3) Prevention of disease and conditions and promotion of well-being (research aimed at the primary prevention of disease, conditions, or ill health or promotion of well-being)
(3.1) Primary prevention interventions to modify behaviors or promote well-being
(3.2) Interventions to alter physical and biological environmental risks
(3.3) Nutrition and chemoprevention
(3.4) Vaccines
(3.5) Resources and infrastructure (prevention)
(4) Detection, screening, and diagnosis (discovery, development, and evaluation of diagnostic, prognostic, and predictive markers and technologies)
(4.1) Discovery and preclinical testing of markers and technologies
(4.2) Evaluation of markers and technologies
(4.3) Influences and impact
(4.4) Population screening
(4.5) Resources and infrastructure (detection)
(5) Development of treatments and therapeutic interventions (discovery and development of therapeutic interventions and testing in model systems and preclinical settings)
(5.1) Pharmaceuticals
(5.2) Cellular and gene therapies
(5.3) Medical devices
(5.4) Surgery
(5.5) Radiotherapy and other noninvasive therapies
(5.6) Psychological and behavioral
(5.7) Physical
(5.8) Complementary
(5.9) Resources and infrastructure (development of treatments)
(6) Evaluation of treatments and therapeutic interventions (testing and evaluation of therapeutic interventions in a clinical, community, or applied settings)
(6.1) Pharmaceuticals
(6.2) Cellular and gene therapies
(6.3) Medical devices
(6.4) Surgery
(6.5) Radiotherapy and other noninvasive therapies
(6.6) Psychological and behavioral
(6.7) Physical
(6.8) Complementary
(6.9) Resources and infrastructure (evaluation of treatments)
(7) Management of diseases and conditions (research into individual care needs and management of disease, conditions, or ill health)
(7.1) Individual care needs
(7.2) End of life care
(7.3) Management and decision-making
(7.4) Resources and infrastructure (disease management)
(8) Health and social care service research (research into the provision and delivery of health and social care services, health policy, and studies of research design, measurements, and methodologies)
(8.1) Organization and delivery of services
(8.2) Health and welfare economics
(8.3) Policy, ethics, and research governance
(8.4) Research design and methodologies
(8.5) Resources and infrastructure (health services)

**Table 2:** The focus of pediatric tuberculosis systematic reviews.

Category and subcategory	Pediatric TB research focus	Proportion (%)
*Aetiology: 4 of *N* = 30 (13.34%)*		
Factors relating to the physical environment	Environmental or external factors associated with the cause, risk, or development of TB disease in children	3/4 (75)
Surveillance and distribution	Mortality in children diagnosed with tuberculosis	1/4 (25)
*Prevention of disease and conditions and promotion of well-being: 4 of *N* = 30 (13.34%)*		
Primary prevention interventions to modify behaviors or promote well-being	Chemoprophylaxis of TB in children	3/4 (75)
Interventions to alter physical and biological environmental risks	Barriers to the implementation of isoniazid preventive therapy for tuberculosis in children	1/4 (25)
*Detection, screening, and diagnosis: 13 of *N* = 30 (43.33%)*		
Discovery and preclinical testing of markers and technologies	Xpert MTB/RIF for diagnosis of TB in children	8/13 (61.5)
Evaluation of markers and technologies	Stool for the diagnosis of TB in children	2/13 (15.4)
Influences and impact	Indeterminate interferon-gamma release assay for the diagnosis of tuberculosis in children	1/13 (7.7)
Population screening	TB screening	2/13 (15.4)
*Development of treatments and therapeutic interventions: 01 of *N* = 30 (3.33%)*		
Pharmaceuticals	Delamanid and bedaquiline to treat multidrug-resistant and extensively drug-resistant tuberculosis in children	1/1 (100)
*Evaluation of treatments and therapeutic interventions: 7 of *N* = 30 (23.33%)*		
Pharmaceuticals	Improve adherence to treatment for pediatric tuberculosis	2/7 (28.5)
Physical	Treatment outcome of TB in children	2/7 (28.5)
Complementary	Childhood tuberculosis treatment outcome and its association with HIV	3/7 (43)
*Management of diseases and conditions: 1 of *N* = 30 (3.33%)*		
Management and decision-making	Hospital management of TB in children	1/1 (100)

Denominator *N* = 30 represents the total number of research focuses identified by all the included reviews. In this case, *N* is greater than the 29 number of included systematic reviews because some reviews had a research focus captured by more than one category. There was no SR on “Underpinning research” and “Health and social care services research”.

**Table 3:** Summary of research priorities identified.

Category and subcategory	Pediatric TB research priority identified	Proportion (%)
*Underpinning research: 06 of *N* = 84 (7.1%)*		
Normal biological development and functioning	Assessing interventions in low- and middle-income countries that explicitly analyze pediatric-inclusive and pediatric-distinct needs and outcomes	2/6 (33.3)
Resources and infrastructure (underpinning)	Comparing the difference in Xpert results if done at, or close to, point of care (for example, in clinics) as compared with in-hospital laboratories for TB diagnosis	1/6 (16.7)
	Assessing close collaboration between clinicians, public health authorities, and field-workers in the management of TB	1/6 (16.7)
	Operational considerations and training strategy in choosing the appropriate collection method for implementation at low health facility level for pediatric TB management	2/6 (33.3)
*Aetiology: 03 of *N* = 84 (3.6%)*		
Research design and methodologies (etiology)	Assessing the use of mixed-method approaches that can assess the pathways linking context-dependent factors with outcomes of TB in children	3/3 (100)
*Prevention of disease and conditions and promotion of well-being: 10 of *N* = 84 (11.9%)*		
Primary prevention interventions to modify behaviors or promote well-being	(i) Assessing the use of IPT in reducing TB-associated morbidity. Assessing the provision of preventive therapy to young children exposed to or infected with tuberculosis	5/10 (50)
Interventions to alter physical and biological environmental risks	Evaluating the prioritization of an IPT-friendly healthcare environment. Providing additional guidance for the use of isoniazid in the prevention of TB in HIV-infected children	4/10 (40)
Vaccines	Evaluating BCG vaccine and HVI status for preventing TB in children	1/10 (10)
*Detection, screening, and diagnosis: 38 of *N* = 84 (45.2%)*		
Discovery and preclinical testing of markers and technologies	(i) How do results with Xpert differ in children with different stages of disease severity, from nonsevere to very severe or disseminated?	3/38 (8)
Evaluation of markers and technologies	Evaluating bacteriological TB diagnostic tests	08/38 (21)
Influences and impact	Evaluating TB diagnosis by improving yield through improvements in specimen collection or preparation (i) Assessing the impact of gene Xpert on patient outcome (e.g., time to diagnosis, time to treatment, disease outcomes, health-system cost, and cost for families)	15/38 (39.5)
	Assessing the rollout of Xpert and its implication on empirical tuberculosis treatment initiation	
	Applying transparent definitions for the certainty of diagnosis (e.g., confirmed tuberculosis and clinical tuberculosis)	
Population screening	(i) Assessing active case-finding for early diagnose of TB in children (ii) Assessing the development of screening algorithms and effective implementation of novel diagnostic tool (iii) Determining the incidence and prevalence of tuberculosis in children	4/38 (10.5)
Resources and infrastructure (detection)	(i) Assessing the specific needs of TB in children, particularly around enhanced infrastructure such as early diagnosis and treatment (ii) Evaluating the promotion of clinical diagnoses and empirical treatment when required (iii) Assessing the role of other respiratory and nonrespiratory specimens (e.g., stool and urine cerebrospinal fluid in the diagnosis of TB in children) (iv) Assessing the role of Xpert in nontraditional tuberculosis settings (e.g., HIV clinics and malnutrition units) (v) Evaluating the challenges of integrating Xpert into the health system	2/38 (5)
		2/38 (5)
		1/38 (3.2)
		3/38 (7.8)
*Development of treatments and therapeutic interventions: 13 of *N* = 84 (15.5%)*		
Pharmaceuticals	Developing treatment for active and latent TB in children	10/13 (76.9)
Cellular and gene therapies	Monitoring of electrolytes (potassium and magnesium) as well as albumin in the management of TB in children	2/13 (15.4)
Resources and infrastructure (development of treatments)	Standardized language to describe barriers to TB treatment initiation, within the TB research and advocacy community	1/13 (7.7)
*Evaluation of treatments and therapeutic interventions: 06 of *N* = 84 (7.1%)*		
Pharmaceuticals	(i) Evaluating the treatment of MDR-TB in children (ii) Evaluating TB/HIV treatment (iii) Assessing the combined use of delamanid and bedaquiline in children	2/06 (33.3)
		1/6 (16.7)
		1/6 (16.7)
Psychological and behavioral	Evaluating IPT treatment of TB in children	2/06 (33.3)
*Management of diseases and conditions: 04 of *N* = 84 (4.8%)*		
Individual care needs	Assessing both patients- and system-level barriers is to improve patient outcomes, especially among young populations	04/04 (100)
*Health and social care service research: 4 of *N* = 84 (4.8%)*		
Organization and delivery of services	Research on improving shorter treatment regimens of TB in children	1/4 (25)
Research designs and methodologies	Development of methods of research assessment and evaluation	1/4 (25)
Resources and infrastructure (health services)	(i) Developing structures, processes, and tools to implement and monitor CCM; health education interventions for HCWs, caregivers, index cases, and the community (ii) Developing a focused approach toward every aspect of child contact management to decrease TB-related morbidity and mortality in children	1/4 (25)
		1/4 (25)

Denominator *N* = 84 represents the total number of research priorities identified by all the included studies.

## Additional Points

*What Is Known about This Topic*. (i) Pediatric TB has been relatively neglected, although recent years have seen a welcome increase in policy focus including the goal of zero childhood tuberculosis deaths. (ii) Advancing a research agenda designed to meet the needs of children is critical to ending the pediatric TB epidemic. (iii) The Stop TB Partnership Child and Adolescent TB Working Group and Treatment Action Group have outlined a detailed list of research priorities for pediatric TB. *What This Study Adds*. (i) A very limited number of systematic reviews and meta-analyses have been published on pediatric TB. This is the first systematic review of pediatric research questions and priorities. (ii) Pediatric TB research focuses mostly on “Detection, Screening and Diagnosis” and “Evaluation of Treatments and Therapeutic Interventions.” (iii) The main areas for pediatric research priorities are on “Detection, Screening and Diagnosis,” “Development of Treatments and Therapeutic Interventions,” and “Prevention of Disease and Conditions, and Promotion of Well-Being.”

## References

[1] World Health Organisation (2020). *World Health Organization. Global tuberculosis control: WHO report 2019. Geneva, Switzerland World Health Organization; 2019*.

[2] World Health Organisation (2013). *The Roadmap for Childhood TB: Toward Zero Deaths*.

[3] World Health Organisation (2018). *Roadmap towards ending TB in children and adolescents, second edition*.

[4] World Health Organisation (2020). *Global Strategy for Tuberculosis Research and Innovation*.

[5] Nicolau I., Ling D., Tian L., Lienhardt C., Pai M. (2012). Research questions and priorities for tuberculosis: a survey of published systematic reviews and meta-analyses. *PLoS One*.

[6] Grant L. R., Hammitt L. L., Murdoch D. R., O’Brien K. L., Scott J. A. (2012). Procedures for collection of induced sputum specimens from children. *Clinical Infectious Diseases*.

[7] Mesman A. W., Soto M., Coit J. (2019). Detection of Mycobacterium tuberculosis in pediatric stool samples using TruTip technology. *BMC Infectious Diseases*.

[8] World Health Organisation (2019). *Global Plan to Stop Tuberculosis 2018-2022*.

[9] TB Working Group (2018). *Research Priorities for Paediatric Tuberculosis*.

[10] Judith G. (2017). *Health Science Literature Review Made Easy. The Matrix Method*.

[11] UK Clinical Research Collaboration (2018). *Health Research Classification System*.

[12] Jafta N., Jeena P. M., Barregard L., Naidoo R. N. (2015). Childhood tuberculosis and exposure to indoor air pollution: a systematic review and meta-analysis. *The International Journal of Tuberculosis and Lung Disease*.

[13] Vecchio A. L., Bocchino M., Lancella L. (2015). Indications to hospital admission and isolation of children with possible or defined Tuberculosis. *Medicine*.

[14] Weaver M. S., Lönnroth K., Howard S. C., Roter D. L., Lam C. G. (2015). Interventions to improve adherence to treatment for paediatric tuberculosis in low- and middle-income countries: a systematic review and meta-analysis. *Bulletin of the World Health Organization*.

[15] D’Ambrosio L., Centis R., Tiberi S. (2017). Delamanid and bedaquiline to treat multidrug-resistant and extensively drug-resistant tuberculosis in children: a systematic review. *Journal of Thoracic Disease*.

[16] Jenkins H. E., Yuen C. M., Rodriguez C. A. (2017). Mortality in children diagnosed with tuberculosis: a systematic review and meta-analysis. *The Lancet Infectious Diseases*.

[17] Sullivan B. J., Esmaili B. E., Cunningham C. K. (2017). Barriers to initiating tuberculosis treatment in sub-Saharan Africa: a systematic review focused on children and youth. *Global Health Action*.

[18] Szkwarko D., Hirsch-Moverman Y., Du Plessis L., Du Preez K., Carr C., Mandalakas A. M. (2017). Child contact management in high tuberculosis burden countries: a mixed-methods systematic review. *PLoS One*.

[19] Charan J., Goyal J. P., Reljic T., Emmanuel P., Patel A., Kumar A. (2018). Isoniazid for the prevention of tuberculosis in HIV-infected children: a systematic review and meta-analysis. *The Pediatric Infectious Disease Journal*.

[20] Faust L., McCarthy A., Schreiber Y. (2018). Recommendations for the screening of paediatric latent tuberculosis infection in indigenous communities: a systematic review of screening strategies among high-risk groups in low-incidence countries. *BMC Public Health*.

[21] Harausz E. P., Garcia-Prats A. J., Law S. (2018). Treatment and outcomes in children with multidrug-resistant tuberculosis: a systematic review and individual patient data meta-analysis. *PLoS Medicine*.

[22] Padmapriyadarsini C., Das M., Burugina Nagaraja S. (2018). Is chemoprophylaxis for child contacts of drug-resistant TB patients beneficial? A systematic review. *Tuberculosis Research and Treatment*.

[23] Sulis G., Amadasi S., Odone A., Penazzato M., Matteelli A. (2018). Antiretroviral therapy in HIV-infected children with Tuberculosis. *The Pediatric Infectious Disease Journal*.

[24] Togun T. O., MacLean E., Kampmann B., Pai M. (2018). Biomarkers for diagnosis of childhood tuberculosis: a systematic review. *PLoS One*.

[25] Grace S. G. (2019). Barriers to the implementation of isoniazid preventive therapy for tuberculosis in children in endemic settings: a review. *Journal of Paediatrics and Child Health*.

[26] Ioos V., Cordel H., Bonnet M. (2019). Alternative sputum collection methods for diagnosis of childhood intrathoracic tuberculosis: a systematic literature review. *Archives of Disease in Childhood*.

[27] MacLean E., Sulis G., Denkinger C. M., Johnston J. C., Pai M., Ahmad Khan F. (2019). Diagnostic accuracy of stool Xpert MTB/RIF for detection of pulmonary tuberculosis in children: a systematic review and meta-analysis. *Journal of Clinical Microbiology*.

[28] Meier N. R., Volken T., Geiger M., Heininger U., Tebruegge M., Ritz N. (2019). Risk factors for indeterminate interferon-gamma release assay for the diagnosis of tuberculosis in children-a systematic review and meta-analysis. *Frontiers in Pediatrics*.

[29] Mesman A. W., Rodriguez C., Ager E., Coit J., Trevisi L., Franke M. F. (2019). Diagnostic accuracy of molecular detection of Mycobacterium tuberculosis in pediatric stool samples: a systematic review and meta-analysis. *Tuberculosis*.

[30] Osman M., Harausz E. P., Garcia-Prats A. J. (2019). Treatment outcomes in global systematic review and patient meta-analysis of children with extensively drug-resistant tuberculosis. *Emerging Infectious Diseases*.

[31] Belay G. M., Wubneh C. A. (2020). Childhood tuberculosis treatment outcome and its association with HIV co-infection in Ethiopia: a systematic review and meta-analysis. *Tropical Medicine and Health*.

[32] Ghosh S., Dronavalli M., Raman S. (2020). Tuberculosis infection in under-2-year-old refugees: should we be screening? A systematic review and meta-regression analysis. *Journal of Paediatrics and Child Health*.

[33] Martinez L., Cords O., Horsburgh C. R. (2020). The risk of tuberculosis in children after close exposure: a systematic review and individual-participant meta-analysis. *The Lancet*.

[34] Seo Y. S., Kang J. M., Kim D. S., Ahn J. G. (2020). Xpert MTB/RIF assay for diagnosis of extrapulmonary tuberculosis in children: a systematic review and meta-analysis. *BMC Infectious Diseases*.

[35] Tola H. H., Khadoura K. J., Jimma W., Nedjat S., Majdzadeh R. (2020). Multidrug resistant tuberculosis treatment outcome in children in developing and developed countries: a systematic review and meta-analysis. *International Journal of Infectious Diseases*.

[36] Gupta R. K., Lucas S. B., Fielding K. L., Lawn S. D. (2015). Prevalence of tuberculosis in post-mortem studies of HIV-infected adults and children in resource-limited settings: a systematic review and meta-analysis. *AIDS*.

[37] Kunkel A., Zur Wiesch P. A., Nathavitharana R. R., Marx F. M., Jenkins H. E., Cohen T. (2016). Smear positivity in paediatric and adult tuberculosis: systematic review and meta-analysis. *BMC Infectious Diseases*.

[38] Pavlinac P. B., Lokken E. M., Walson J. L., Richardson B. A., Crump J. A., John-Stewart G. C. (2016). Mycobacterium tuberculosis bacteremia in adults and children: a systematic review and meta-analysis. *The international Journal of Tuberculosis and Lung Disease*.

[39] Lyu M., Zhou J., Fang T., Fu T., Cheng Y. (2019). Which types of sample is better for Xpert MTB/RIF to diagnose adult and pediatrics pulmonary tuberculosis: a systematic review and meta-analysis. *Clinica Chimica Acta*.

[40] Guyatt G. H., Guyatt G. H., Oxman A. D. (2008). GRADE: an emerging consensus on rating quality of evidence and strength of recommendations. *BMJ*.

[41] Villanueva P., Neth O., Ritz N., Tebruegge M. (2018). Use of Xpert MTB/RIF ultra assays among paediatric tuberculosis experts in Europe. *European Respiratory Journal*.

[42] Furin J. (2019). Advances in the diagnosis, treatment, and prevention of tuberculosis in children. *Expert Review of Respiratory Medicine*.

[43] WHO (2016). *Chest Radiography in Tuberculosis Detection-Summary of Current who Recommendaitons and Guideline on Programmatic Approaches*.

[44] Benabdellah A., Taleb-Bendiab R., Sour N., Allal-Taouli K. (2016). *Recent research and review studies for“tuberculosis”*.

[45] Nicolau I., Ling D., Tian L., Lienhardt C., Pai M. (2013). Methodological and reporting quality of systematic reviews on tuberculosis. *The International Journal of Tuberculosis and Lung Disease*.

